# Induction of intracellular ferritin expression in embryo-derived *Ixodes scapularis* cell line (ISE6)

**DOI:** 10.1038/s41598-018-34860-3

**Published:** 2018-11-08

**Authors:** Emmanuel Pacia Hernandez, Kodai Kusakisako, Melbourne Rio Talactac, Remil Linggatong Galay, Kentaro Yoshii, Tetsuya Tanaka

**Affiliations:** 1 Laboratory of Infectious Diseases, Joint Faculty of Veterinary Medicine, Kagoshima University, 1-21-24 Korimoto, Kagoshima, 890-0056 Japan; 2 Department of Pathological and Preventive Veterinary Science, The United Graduate School of Veterinary Science, Yamaguchi University, Yoshida, Yamaguchi 753-8515 Japan; 3 Department of Clinical and Population Health, College of Veterinary Medicine and Biomedical Sciences, Cavite State University, Cavite, 4122 Philippines; 4 Department of Veterinary Paraclinical Sciences, University of the Philippines Los Baños, College, Laguna 3004 Philippines; 5 Laboratory of Public Health, Faculty of Veterinary Medicine, Hokkaido University, Sapporo, Hokkaido 060-0818 Japan

## Abstract

Iron is a very important nutrient for cells; however, it could also cause fatal effects because of its capability to trigger oxidative stress. Due to high exposure to iron from their blood diet, ticks make use of several mechanisms to cope up with oxidative stress. One mechanism is iron sequestration by ferritin and its control protein (IRP). Since the IRP activity is dependent on the ferrous iron concentration, we tried to induce intracellular ferritin (FER1) protein expression by exposing *Ixodes scapularis* embryo-derived cell line (ISE6) to different concentrations of ferrous sulphate at different time points. We were able to induce FER1 protein after exposure to 2 mM of ferrous sulphate for 48 h, as observed in both Western blotting and indirect immunofluorescent antibody tests. This could indicate that the FER1 produced could be a product of the release of IRPs from the *FER1* mRNA leading to its translation. The RNA interference of FER1, through the transfection of dsRNA, led to an increase in mortality and decrease in the cellular proliferation of ISE6 cells. Overall, ISE6 cells could be a good tool in further understanding the mechanism of FER1 action, not just in *Ixodes* ticks but in other tick species as well.

## Introduction

Iron is vital to life, for it is important in many metabolic processes of the cells, including oxygen transport and deoxyribonucleic acid (DNA) synthesis, as well as electron transport^1^. On the other hand, excess iron specifically in the ferrous ion (Fe^2+^) could lead to deleterious effects due to its ability to trigger the Fenton reaction. The Fenton reaction is a result of iron reacting to hydrogen peroxide (H_2_O_2_), resulting in the generation of hydroxyl radicals. Thus, iron must be carefully balanced in cells^2^.

Ticks are obligate blood-feeding arthropods. Since tick digestion occurs within the digestive cells, they are more exposed to increased amounts of iron coming from the host blood as compared to other blood-feeding arthropods. Thus, ticks make use of several strategies to control iron levels^3^. One strategy ticks utilise is iron sequestration. Several proteins have been shown to be important in the sequestration of iron. These include two ferritins, such as intracellular ferritin (FER1) and secretory ferritin (FER2), and they also include iron regulatory proteins (IRP) to control FER1 expression^4^.

Ferritins are iron-storage proteins found in almost all organisms. The primary function of FER is to store excess iron available in the cellular iron pool. The iron storage process involves the binding and oxidation of Fe^2+^ and the formation of ferric ion (Fe^3+^) in the core cavity^3^. FER1 protein expression is regulated by the interaction between IRPs and iron-responsive elements (IRE) in the *FER1* mRNA. Thus, these interactions are dependent on the cell’s iron availability. During periods of low iron levels, IRP binds to the IRE element in the untranslated region of the *FER1* mRNA, effectively blocking protein translation. When iron levels increase, Fe-S clusters can form an insert themselves into tick IRPs; the IRPs ar then converted into aconitase and detach from the mRNA iron loop. This results in FER1 translation so that newly synthesised FER1 can sequester the free iron to protect the tick cell from oxidative stress^4,5^.

Tick cell lines have been used in the study of pathogenic organisms that can be transmitted by ticks^6^. Recently, studies involving immunology and physiology as well as response to oxidative stress utilised tick cell lines^6,7^; for this purpose, the embryo-derived tick cell line from *Ixodes scapularis* (ISE6) is one of the most used tick cell lines. Despite ISE6 cells being widely used, its protein composition still remains unknown. Researchers have attempted to define the origin of ISE6 cells, but were only able to establish that these cells have a neuron-like phenotype while retaining some proteomic characteristics similar to those of another embryo-derived cell line^6^.

Since ISE6 cells are known to be embryo derived^6^, we hypothesised that they retain certain characteristics of embryonic tissues. In previous studies on *H. longicornis* embryonic tissue, detection was possible for *FER1* mRNA but not the FER1 protein, and for FER2 protein but not *FER2* mRNA^8^. Therefore, this study would like to establish a method to induce FER1 protein expression in ISE6 cells to be used for further understanding the mechanism of iron regulation *in vitro* in ticks.

## Results

### Identification of ferritin and IRP genes of ISE6

**Figure 1:**
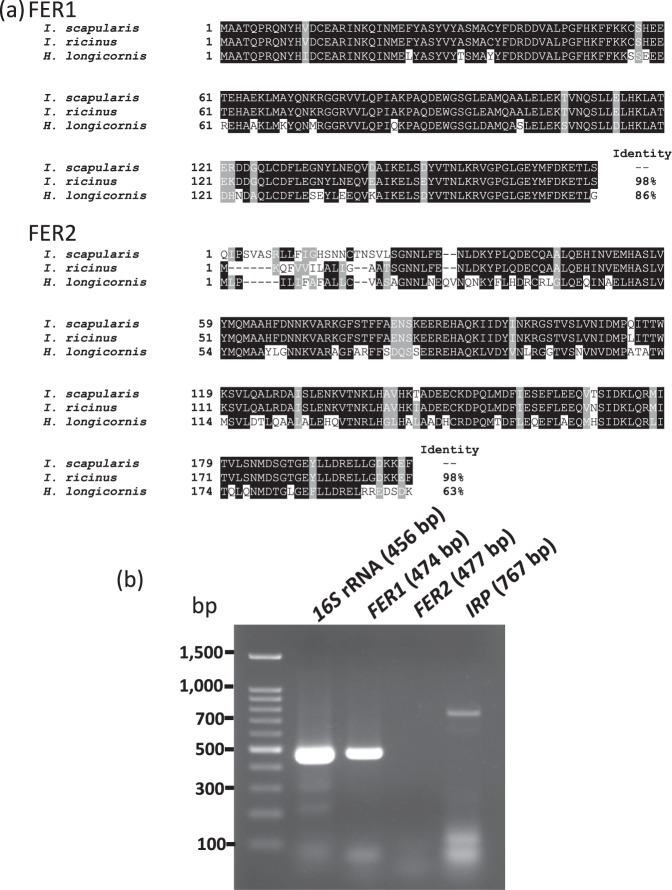
Identification of the ferritin protein and genes of ISE6 cells. (**a**) Multiple sequence alignment of the amino acid sequences of FER1 and FER2 of *Ixodes scapularis* with *Ixodes ricinus* and *Haemaphysalis longicornis* ferritins. Identical residues are shaded black, while similar residues are shaded gray. The percent identities with FER1 and FER2 are placed at the end of the sequences. The GenBank accession numbers of ferritin sequences are as follows: *I. scapularis* FER1 (AAQ54714.1), *I. scapularis* FER2 (XP_002415446.1), *I. ricinus* FER1 (AAC19131.1), *I. ricinus* FER2 (ACJ70653.1), *H. longicornis* FER1 (AAQ54713.1), and *H. longicornis* FER2 (BAN13552.1). (**b**) RT-PCR of ISE6 cells for *FER1*, *FER2*, and *IRP*. Total RNA was extracted from ISE6 cells. cDNA was synthesised and subjected to RT-PCR. PCR products were run on 1.5% agarose gel and stained with ethidium bromide. *16S* rRNA was used as a loading control. The PCR result are representatives of three separate experiments.

Identified *H. longicornis* and *I. ricinus* ferritins and IRPs were subjected to protein BLAST analysis to identify their homologues in *I*. *scapularis*. The BLAST analysis revealed that *H. longicornis* FER1 (Accession No. AAQ54713.1) has 86% identity, and *I. ricinus* FER1 (Accession No. AAC19131.1) has 98% identity with *I. scapularis* FER1 (Accession No. AAQ54714.1) (Fig. 1a). *H. longicornis* FER2 (Accession No. BAN13552.1) has 63% identity, while *I. ricinus* FER2 (Accession No. ACJ70653.1) has 98% identity with *I. scapularis* FER2 (Accession No. XP_002415446.1) (Fig. 1a). Using the NCBI database, the gene sequences of the predicted *I. scapularis* ferritins were determined, and specific primers were designed to detect *I. scapularis* ferritin in ISE6 cells. The expected 474 base pairs (bp) band corresponding to *FER1* mRNA was detected, while the expected 477 bp band corresponding to *FER2* mRNA was not observed (Fig. 1b). For further confirmation of the absence of *FER2* mRNA, another RT-PCT was performed using a different set of primers (ISFER2 RT forward B and ISFER2 RT reverse B), and no bands were observed (data not shown). The sequence of *IRP1* (EU885952) from *I. ricinus*, a tick closely related to *I. scapularis*, was used to design primers for *IRP* mRNA detection in which the expected 767 bp band corresponding to *IRP* was detected on ISE6 cell (Fig. 1b).

### FER1 proteins are induced in ISE6 cells exposed to 2 mM ferrous sulphate

**Figure 2:**
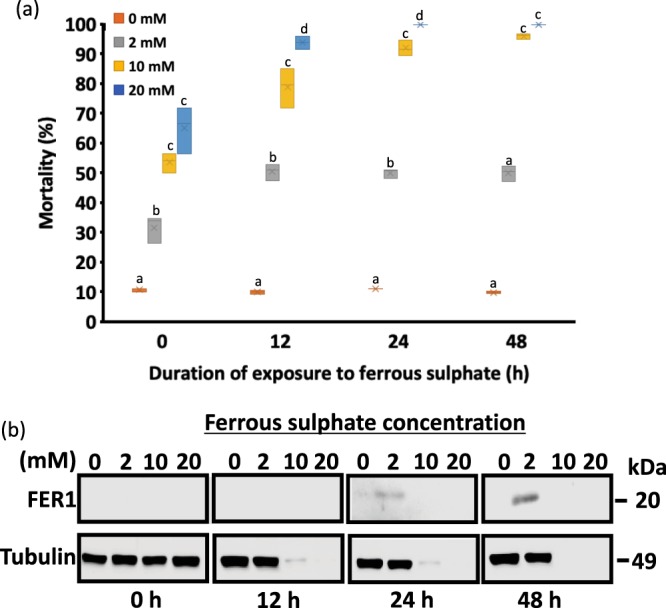
(**a**) Box and whisker plot showing the distribution of mortality in percentage of ISE6 cells exposed to different concentrations of ferrous sulphate. Two hundred fifty microliters of 1 × 10^6^ cells/ml was transferred to a 48-well plate and incubated overnight. The next day, the media were replaced by ferrous sulphate–enriched media at different concentrations (0, 2, 10, and 20 mM). Mortality was checked at different time points (0, 12, 24, and 48 h) using Trypan blue stain. A one-way ANOVA with Bonferonni multiple comparison tests was performed (Tables S4 and S3). Time points with the same letter are not significantly different at *P* < 0.05. (**b**) Western blotting of ISE6 cells exposed to different concentrations of ferrous sulphate at different time points. Protein analysis of FER1 was observed using Western blotting. Anti-mouse tubulin was used as a loading control. Full length blots are presented in Fig. S4. The Western blotting results are representatives of three separate experiments. The data presented are results of three independent experiments with the Trypan blue staining performed in two technical replicates for each experiment.

To induce FER1 expression via the removal of IRP from the *FER1* mRNA, cells were exposed to high concentrations of Fe^2+^ through the addition of ferrous sulphate. In deciding an experimental concentration of ferrous sulphate for ISE6 cells, Trypan blue assay was performed to check for cellular mortality of cells treated with several concentrations of ferrous sulphate (0, 2, 10, and 20 mM) at different time points (0, 12, 24, and 48 h) (Fig. 2a; Table 1). The Trypan blue assay showed that even immediately after the addition of ferrous sulphate, the survival of cells was affected. Concentrations of 10 and 20 mM of ferrous sulphate caused the mortality of almost all cells. This result could indicate that an increased level of ferrous sulphate in a cell could cause cellular toxicity. Western blotting of cell lysates after exposure to increasing concentrations of ferrous sulphate at different time points was also performed (Fig. 2b). An approximately 20 kDa band that corresponds to the FER1 protein was faintly detected at 24 h but clearly seen after 48 h of exposure to 2 mM of ferrous sulphate. No bands at approximately 49 kDa corresponding to tubulin, as well as FER1, were observed from 12 h to 48 h in 10 and 20 mM of ferrous sulphate–exposed cells because almost all the cells had already died. The maximum concentration of 2 mM of ferrous sulphate for 48 h was therefore used in the succeeding experiments.

**Table 1:** Mortality (%) of ISE6 cells exposed to different concentrations of ferrous sulphate at different time points.

Ferrous sulphate concentration (mM)	Time of Exposure (h)			
	0	12	24	48
0	10.73 ± 0.578	9.91 ± 0.671	11.91 ± 0.064	9.89 ± 0.413
2	31.68 ± 4.555	50.53 ± 2.815	50.04 ± 1.510	50.05 ± 2.597
10	53.60 ± 3.357^a^	78.91 ± 6.714	92.05 ± 2.554	96.13 ± 0.933^a^
20	65.06 ± 7.795^a^	93.95 ± 2.245	100.00 ± 0.000	100.00 ± 0.000^a^

Data are presented as mean ± S.D. The results for the normality and homogeneity are presented in Table S1. A one-way ANOVA with Bonferonni multiple comparison test was performed (Tables S2 and S3). Statistical significance was set as ^*^P < 0.05. Columns with the same superscript are not significantly different at *P* < 0.05. The data presented are results of three separate experiments with two technical replicates for each experiment.

**Figure 3:**
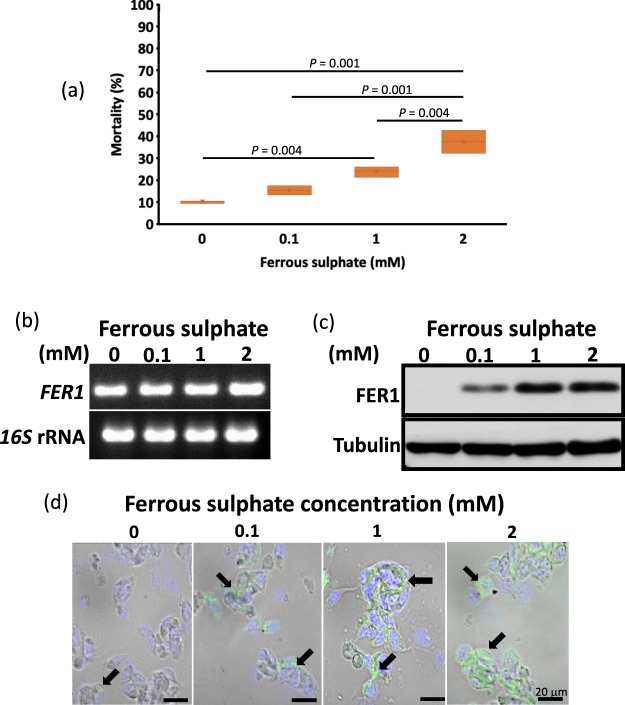
(**a**) Box and whisker plot showing the distribution of mortality in percentage of ISE6 cells exposed to different concentrations of ferrous sulphate for 48 h. Two hundred and fifty microliters of 1 × 10^6^ cells/ml was transferred to a 48-well plate and incubated overnight. The next day, the media were replaced by ferrous sulphate–enriched media at different concentrations (0, 0.1, 1, and 2 mM). Mortality was checked after 48 h using Trypan blue stain. A one-way ANOVA with Bonferonni multiple comparison tests was performed (Tables S5 and S6). (**b**) *FER1* mRNA expression of ferrous sulphate (0, 0.1, 1, and 2 mM)–exposed cells for *FER1* was observed using RT-PCR. *16S* rRNA was used as a loading control. Full length gels are presented in Fig. S5. The RT-PCR result is representative of three separate experiments. (**c**) FER1 protein expression of ferrous sulphate (0, 0.1, 1, and 2 mM)–exposed cells for FER1 was observed using Western blotting. Anti-mouse tubulin was used as a loading control. Full length blots are presented in Fig. S2. The Western blotting result is representative of three separate experiments. (**d**) Localization of FER1 protein was observed using IFAT. Arrowheads indicate positive fluorescence. The IFAT result is representative of three separate experiments. Bar = 10 μm. The data presented are results of three independent experiments with the Trypan blue staining performed in two technical replicates for each experiment.

To be able to determine the effect of lower doses of ferrous sulphate on ISE6 survival and the expression of FER1, ISE6 cells were exposed to lower doses (0, 0.1, 1, and 2 mM) of ferrous sulphate for 48 h. A dose-dependent increase in mortality was observed (Fig. 3a; Table 2).

**Table 2:** Mortality (%) of cells exposed to different concentrations of ferrous sulphate at 48 h.

Ferrous sulphate concentration (mM)			
0	0.1	1	2
10.16 ± 0.622^a^	15.47 ± 2.081^ab^	23.99 ± 2.433^b^	37.56 ± 5.270

Data are presented as mean ± S.D. The results for the normality and homogeneity are presented in Table S4. A one-way ANOVA with Bonferonni multiple comparison test was performed (Tables S5 and S6). Statistical significance was set as ^*^P < 0.05. Columns with the same superscript are not significantly different at *P* < 0.05. The data presented are results of three separate experiments with two technical replicates for each experiment.

To be able to determine whether FER1 protein expression is transcriptionally regulated, RT-PCR was performed, which showed that the *FER1* mRNA of ferrous sulphate–exposed cells did not show any difference in band intensity (Fig. 3b). On the other hand, dose-dependent FER1 expression was also observed with Western blotting (Fig. 3c). FER1 expression in cells exposed to ferrous sulphate for 48 h was further confirmed using an indirect immunofluorescent antibody test (IFAT) (Fig. 3d). Positive signals for FER1 in the cytoplasm were more intense in ferrous sulphate–exposed cells as compared to non-exposed cells. These results show that the FER1 protein of ISE6 cells could be expressed consistently when exposed to 2 mM ferrous sulphate for 48 h. The results could also indicate that the expressed FER1 is post-transcriptionally regulated.

### Silencing of the *FER1* gene increases the intracellular ferrous iron concentration in ISE6 cells

**Figure 4:**
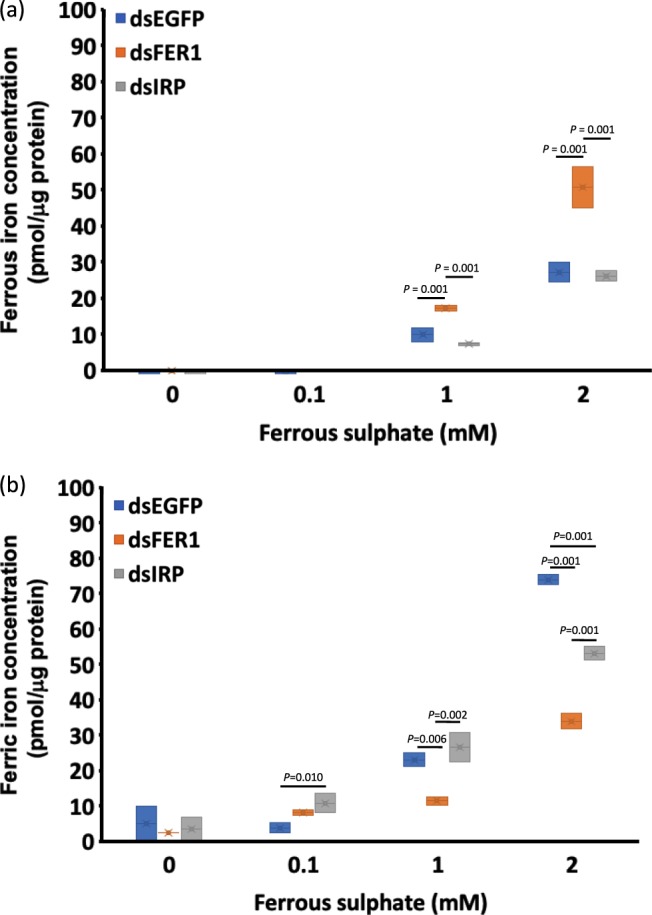
(**a**) Box and whisker plot showing the distribution of ferrous iron concentration of knocked down ISE6 cells exposed to different concentrations of ferrous sulphate as determined by ferrozine assay. Cleared lysates from knocked down ISE6 cells exposed to different concentrations of ferrous sulphate, and the concentration was determined using a Micro BCA Protein Assay Kit. Ferrous iron was determined using a ferrozine assay in the absence of a reducing agent. A one-way ANOVA with Bonferonni multiple comparison tests was performed (Tables S8 and S9) (**b**) Box and whisker plot showing the distribution of ferric iron concentration of knocked down ISE6 cells exposed to different concentrations of ferrous sulphate as determined by ferrozine assay. Cleared lysates from knocked down ISE6 cells exposed to different concentrations of ferrous sulphate, and the concentration was determined using a Micro BCA Protein Assay Kit. Ferric iron was determined using the ferrozine assay by determining the difference between the total and ferrous iron concentrations. A one-way ANOVA with Bonferonni multiple comparison tests was performed (Tables S11 and S12). The data presented are results of three independent experiments with the ferrozine assay performed in two technical replicates for each experiment.

To determine the effect on the ferrous and ferric ion concentrations in *FER1* and *IRP* knockdown cells, ferrozine assay was performed on ferrous sulphate-enriched cells. *FER1* and *IRP* knockdown ISE6 cells were exposed to different concentrations of ferrous sulphate (0, 0.1, 1, and 2 mM) and then subjected to the assay. In this assay, ferrous iron concentration was significantly greater in *FER1* knockdown cells exposed to 1 and 2 mM than in the control, enhanced green fluorescent protein (*EGFP*) gene, and *IRP* knockdown cells (Fig. 4a; Table 3). Conversely, ferric iron concentration was significantly lower in *FER1* knockdown cells exposed to ferrous sulphate at 1 and 2 mM than in *EGFP* and *IRP* knockdown cells (Fig. 4b; Table 4). These results are consistent with a role of FER1 in the conversion of Fe^2+^ to Fe^3+^. They also demonstrate that the induced FER1 protein is functional in terms of storing iron.

**Table 3:** Ferrous iron concentration (pmol/ μg protein) of knockdown cells exposed to different concentrations of ferrous sulphate at 48 h.

Knockdown group	Ferrous sulphate concentration (mM)			
	0	0.1	1	2
dsEGFP	−5.02 ± 5.016	−1.39 ± 1.388	9.97 ± 1.946^a^	27.31 ± 0.413^a^
dsFER	0.00 ± 0.000	−3.87 ± 0.829	17.30 ± 0.872	50.83 ± 5.801
dsIRP	−3.41 ± 3.409	−4.22 ± 2.713	7.36 ± 0.420^a^	26.25 ± 1.529^a^

Data are presented as mean ± S.D. The results for the normality and homogeneity are presented in Table S7. A one-way ANOVA with Bonferonni multiple comparison test was performed (Tables S8 and S9). Statistical significance was set as ^*^P < 0.05. Columns with the same superscript are not significantly different at *P* < 0.05. The data presented are results of three separate experiments with two technical replicates for each experiment.

**Table 4:** Ferric iron concentration (pmol/μg protein) of knockdown cells exposed to different concentrations of ferrous sulphate at 48 h.

Knockdown group	Ferrous sulphate concentration (mM)			
	0	0.1	1	2
dsEGFP	5.02 ± 5.016	3.91 ± 1.388^a^	23.10 ± 1.945^a^	74.05 ± 1.388
dsFER	2.57 ± 0.000	8.29 ± 0.829^ab^	11.48 ± 1.163	33.98 ± 2.210
dsIRP	3.41 ± 3.409	10.85 ± 2.713^b^	26.70 ± 4.204^a^	53.27 ± 2.039

Data are presented as mean ± S.D. The results for the normality and homogeneity are presented in Table S10. A one-way ANOVA with Bonferonni multiple comparison test was performed (Tables S11 and S12). Statistical significance was set as ^*^P < 0.05. Columns with the same superscript are not significantly different at *P* < 0.05. The data presented are results of three separate experiments with two technical replicates for each experiment.

### Silencing of the *FER1* gene resulted in increased cellular mortality and decreased cellular proliferation

**Figure 5:**
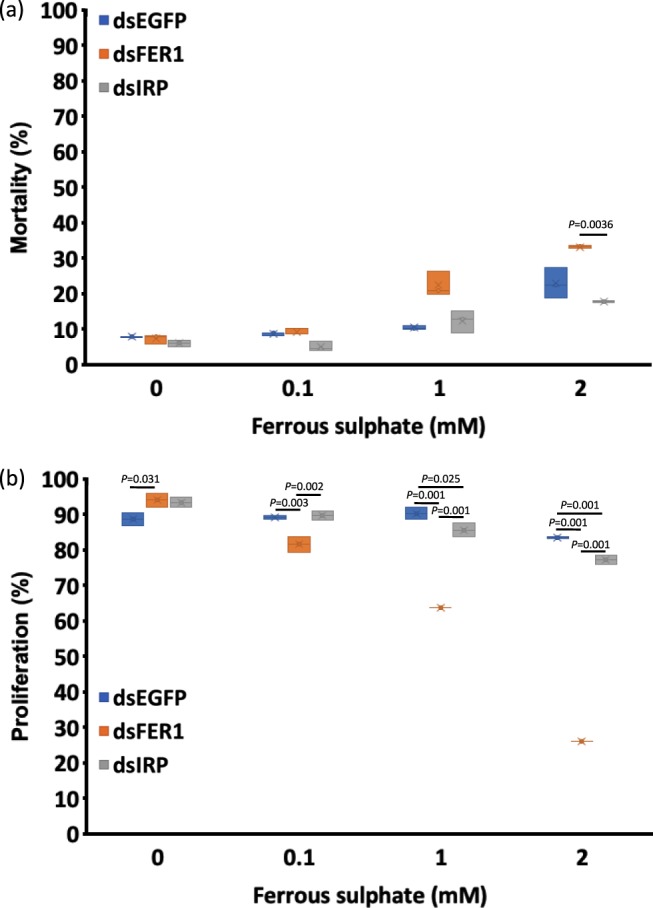
ISE6 cells were exposed to 800 ng of dsRNA for 48 h and transfected using HilyMax. Two hundred and fifty microliters of 1 × 10^6^ silenced ISE6 cells/ml was transferred to a 48-well plate and incubated overnight. The next day, the media were replaced by iron-enriched media at different concentrations of 0, 0.1, 1, and 2 mM ferrous sulphate. (**a**) Box and whisker plot showing the distribution of the mortality of the ISE6 cells in percentage using Trypan blue stain at different time points. A Kruskall –Wallis test with Bonferonni multiple comparison tests was performed to analyse the mortality data (Tables S14 and S15). (**b**) Box and whisker plot showing the distribution of the proliferation rate in percentage of ISE6 cells using MTT assay at different time points. A one-way ANOVA with Bonferonni multiple comparison tests was performed to analyse the proliferation rate data (Tables S17 and S18). The data presented are results of three independent experiments with the Trypan blue and MTT assays performed in two technical replicates for each experiment.

Ferritin proteins have already been demonstrated to be important in tick survival and reproduction^4,8^. Therefore, to investigate whether it would reflect on ISE6 cells, RNAi was conducted to determine whether *FER1* and *IRP* gene silencing would affect the survival and proliferation of ISE6 cells. Gene silencing was confirmed by RT-PCR (Fig. S1). Knockdown of the *FER1* gene with subsequent exposure to ferrous sulphate was investigated in Trypan blue and MTT assays. In the Trypan blue assays, with exposure to ferrous sulphate at 2 mM, *FER1* knockdown cells showed apparently greater mortality than *EGFP* knockdown cells and significantly greater mortality than *IRP* knockdown cells (Fig. 5a; Table 5). In the MTT assays, with exposure to ferrous sulphate from 0.1 to 2 mM, *FER1* knockdown cells showed significantly lower cellular proliferation than *EGFP* and *IRP* knockdown cells (Fig. 5b; Table 6). This could indicate the protective capability of FER1 during periods of iron overload.

**Table 5:** Mortality (%) of knockdown cells exposed to different concentrations of ferrous sulphate at 48 h

Knockdown group	Ferrous sulphate concentration (mM)			
	0	0.1	1	2
dsEGFP	7.83 ± 0.117	8.84 ± 0.437	10.49 ± 0.569	23.01 ± 4.256^a^
dsFER	7.41 ± 1.329	9.20 ± 0.995	22.39 ± 3.491	33.27 ± 0.379
dsIRP	6.06 ± 0.924	5.16 ± 1.326	12.40 ± 3.086	17.79 ± 0.252^a^

Data are presented as mean ± S.D. The results for the normality and homogeneity are presented in Table S13. A Kruskall-Wallis test with Bonferonni multiple comparison test was performed (Tables S14 and S15). Statistical significance was set as ^*^P < 0.05. Columns with the same superscript are not significantly different at *P* < 0.05. The data presented are results of three separate experiments with two technical replicates for each experiment.

**Table 6:** Proliferation (%) of knockdown cells exposed to different concentrations of ferrous sulphate at 48 h.

Knockdown group	Ferrous sulphate concentration (mM)			
	0	0.1	1	2
dsEGFP	88.77 ± 1.854^a^	89.19 ± 0.515^a^	90.42 ± 1.751	83.68 ± 0.257
dsFER	94.18 ± 2.008^b^	81.67 ± 2.163	63.95 ± 0.000	26.11 ± 0.051
dsIRP	93.56 ± 1.493^ab^	89.91 ± 1.339^a^	85.79 ± 1.854	77.34 ± 1.236

Data are presented as mean ± S.D. The results for the normality and homogeneity are presented in Table S16. A one-way ANOVA with Bonferonni multiple comparison test was performed (Tables S17 and S18). Statistical significance was set as ^*^P < 0.05. Columns with the same superscript are not significantly different at *P* < 0.05. The data presented are results of three separate experiments with two technical replicates for each experiment.

## Discussion

Ticks are obligate blood-feeding ectoparasites known to be vectors of several diseases that are important to health and the economy^9^. Until now, the control of these ectoparasites has relied mainly on the use of chemical acaricides^10^. One of the methods being considered to control these arthropods is through the development of anti-tick vaccines^11^. FER1 is one candidate molecule being considered for a tick vaccine^12^. Therefore, a thorough understanding of FER1 is warranted; however, tick experiments regarding FER1 always must consider the presence of FER2 and the basal FER1 protein level^4,8^. In this regard, the embryo-derived ISE6 cell line is an ideal candidate because of the absence of the FER1 protein and *FER2* mRNA on its natural state, as observed in the present study. Although Oliver *et al*. showed that ISE6 cells differentiate towards a predominant neuron-like phenotype, their studies also demonstrated ISE6 cells can maintain certain proteomic characteristics due to their embryonic origin^6^. As cells possessing embryonic characteristics, this cell line could be used for elucidating both the function and regulation of FER1.

Ferritin has been reported to be induced by several compounds, such as prostaglandins, oxalomalate, or hydrogen peroxide^13–15^. In the present study, we have tried to induce FER1 expression in ISE6 cells by increasing the concentration of ferrous sulphate in the medium; in addition, the toxic effect of iron caused high mortality in ISE6 cells (Fig. 2a; Table 1) when concentrations of iron were too high—10 to 20 mM. The immediate mortality with exposure to ferrous sulphate at high concentrations could represent accidental cell death (ACD). ACDs are usually caused by physical, mechanical, or chemical insults^16^. The cause of the ACDs in this study was suggested to be chemical insult because ferrous sulphate-enriched media tends to be acidic (pH 5.0), thus inducing change in intracellular pH.

High mortality was also observed in 2 mM ferrous sulphate–exposed tick cells (Fig. 2a; Table 1). The addition of ferrous sulphate has resulted in increased ferrous iron concentration in the cellular environment^17^. Ferrous ions are more toxic than ferric ions because when a ferrous ion reacts to H_2_O_2_ through the Fenton reaction, it could result in the generation of hydroxyl radicals. Highly reactive hydroxyl radicals could cause oxidative stress, such as lipid peroxidation, DNA strand breaks, and the degradation of other molecules^5,18^. In contrast with the ACD noted at high concentrations of ferrous sulphate, this cell mortality could be classified as iron-dependent regulated cell death (RCD), ferroptosis^19^. In ferroptosis, the iron-catalyzed lipid peroxidases accumulates to toxic levels due to the inactivation of the phospholipid peroxidase^16,19^. Although ferroptosis has not been established in ticks, ferroptotic death can be hypothesized as the cause of ISE6 cell mortality, and this hypothesis warrants further investigation in biochemical tests.

After 12 h of exposure, mortality did not appear to increase anymore (Fig. 2a; Table 1). This could indicate that cells have developed a protective mechanism to cope with the increased oxidative stress, possibly by the sequestration of the ferrous iron molecule. This protective mechanism could be in the form of the increased transcription of anti-oxidative stress molecules, protein trafficking, or vacuolar function^20^. This is said to be an antioxidant response^21^.

Western blotting and IFAT results have shown that at 48 h, the intracellular iron storage protein, FER1, has been observed (Fig. 3c,d). This phenomenon is consistent with a previous theory on the role of IRP and FER in the sequestration of free iron to protect tick cells from oxidative stress^4,5^. RT-PCR results have also shown that the mRNA level of ferritin did not greatly vary (Fig. 3b). However, a more accurate test such as quantitative real-time RT-PCR could directly measure the change in the mRNA transcription level. Nevertheless, the RT-PCR results could still indicate that the produced ferritin is post-transcriptionally regulated specifically by IRP-IRE interactions^22^.

It takes time to express FER1 after cells are exposed to inducers. In a previous experiment on mouse cell lines, FER was induced by exposure to H_2_O_2_. The translational control of IRP peaked at 1 h and continually elevated until after 2 h, before the activity of the IRP decreased for the *FER* mRNA to be available for translation. Synthesis of FER starts at 4 h and continually increased for up to 24 h^23^. The time-dependent cytoprotective mechanism was also observed in porcine endothelium cells, wherein ferritin expression was not observed before 16 h and could protect cells^24^. Experiments in inducing FER expression by oxalomalate indicated the induction of ferritin in human, rat, and mouse cell lines beginning 48 h after exposure^25^. It is also interesting to note that the slow feeding phase in ticks takes 6 to 9 days for *Ixodes* ticks^26^. This feeding phase may perhaps be a mechanism for protecting ticks from iron overload by the intake of iron just sufficient to trigger FER1 translation. During the rapid feeding phase and intracellular digestion, FER1 protein would be readily available. However, further experiments are needed to prove this hypothesis.

Knockdown of *FER1* and exposure to 1 or 2 mM of ferrous sulphate in ISE6 cells resulted in increased ferrous iron concentrations and decreased ferric iron concentrations in cells as observed in the ferrozine assay (Fig. 4; Tables 3,4). These results could indicate that the induced FER1 is functionally capable of converting Fe^2+^ to a Fe^3+^ state^27^. Since FER1 had not been induced in a *FER1* knockdown, the amount of Fe^2+^ increased; thus, the ability to induce oxidative stress also increased.

With exposure to 2 mM ferrous sulphate, *FER1* knockdown yielded apparently greater mortality than *EGFP* knockdown and significantly greater mortality than *IRP* knockdown (Fig. 5a; Table 5). This is a further indication of the time dependency for FER1 translation due to the presence of IRP. Accordingly, FER1 may already have been present in *IRP* knockdown cells when they were exposed to ferrous sulphate, thus protecting the cells and explaining the findings of lower mortality. In previous experiments using actual ticks, the knockdown of *FER1* also resulted in decreased survival and reproduction when exposed to increased iron, such as during blood feeding^4,8^. On the other hand, the knockdown of *IRP* resulted in decreased mortality of ISE6 cells at lower doses of ferrous sulphate (Fig. 5a; Table 5).

With exposure to 0.1 to 2 mM ferrous sulphate, *FER1* knockdown also yielded lower cellular proliferation than *EGFP* and *IRP* knockdowns, providing a further indication of the cytoprotective capability of FER1 (Fig. 5b; Table 6). Since FER1 is present in *EGFP* and *IRP* knockdown cells, they have greater protection from oxidative stress due to increased ferrous iron^18^.

This evidence further strengthens the hypothesis that the cell death at 2 mM ferrous sulphate exposure is RCD, such as ferroptosis; ACD, which cannot be prevented or modulated and therefore cannot be targeted by interventions, RCD involves molecular mechanisms that can be targets for intervention^18,19^. In this case, the alteration could have occurred when lipid peroxidation was suppressed by the expression of FER1, which in turn sequestered the iron molecules.

In conclusion, we were able to induce the expression of the FER1 protein in the *I. scapularis* embryo-derived cell line (ISE6) after exposure to 2 mM of ferrous sulphate for 48 h. We have also shown that the FER1 induced using this method has ferroxidase activity similar to the FER1 in ticks. This study could be useful in understanding the iron metabolism in ticks, and the induction of intracellular ferritin, and represent a potentially major contribution to research on this molecule.

## Methods

### Culture of cells

The ISE6 cell line from the embryo of *Ixodes scapularis* was grown at 34 °C in L-15B medium (pH 6.4–6.6) with 10% fetal bovine serum (FBS) (Biosera, Dominican Republic), 5% tryptose phosphate broth, and antibiotics^28,29^.

### Identification of the *Ferritin* gene of ISE6 cells

BLAST analysis (https://blast.ncbi.nlm.nih.gov/Blast.cgi) of *H. longicornis* ferritin and *I. ricinus* IRP was used to determine their homologue in *I*. *scapularis*. Using the NCBI database, we determined the gene sequence of the predicted *I. scapularis* ferritins and IRP. From the gene sequence, gene-specific detection primers were designed: forward primers (ISFer1 RT-F, ISFer2 RT-F, IRP RT-F) and reverse primers (ISFer1 RT-R, ISFer2 RT-R, IRP RT-R) (Table 7).

**Table 7:** Gene-specific primers used in this study.

Primers	Sequence [5′ → 3′]
ISFER1 RT forward	ACTGCGAAGCTCGCATCAACAA
ISFER1 RT reverse	ACAGGGTCTCCTTGTCGAACAT
ISFER2 RT forward	TTGCAAGCGCTGCGAGATGC
ISFER2 RT reverse	AGGCCCCTGCTCCCCAGAAT
ISFER2 RT forward B	ACAAAGTGGCACGCAAAGGT
ISFER2 RT reverse B	AAAACTCCTTCTTGTCCCCGAGCA
ISFER1 RNAi forward	TTGCAGCAGAAAACAGCCCTTG
ISFER1 RNAi reverse	TTGTCGAACATGTACTCTCCCAG
ISFER1 T7 forward	*TAATACGACTCACTATAGG*TTGCAGCAGAAAACAGCCCTTG
ISFER1 T7 reverse	*TAATACGACTCACTATAGG*TTGTCGAACATGTACTCTCCCAG
ISIRP RT forward	GCATGCATTGAAGATGCCGT
ISIRP RT reverse	GGAAGTAGGCCAACTCGACC
ISIRP RNAi forward	TCGACGTACATCAAGTGCCC
ISIRP RNAi reverse	GGAAGTAGGCCAACTCGACC
ISIRP T7 forward	*TAATACGACTCACTATAGG* TCGACGTACATCAAGTGCCC
ISIRP T7 reverse	*TAATACGACTCACTATAGG* GGAAGTAGGCCAACTCGACC
16S RT forward	CTGCTCAATGATTTTTTAAATTGCTGTGG
16S RT reverse	CCGGTCTGAACTCAGATCAAGTA
EGFP RNAi forward	GACGTAAACGGCCACAAGTT
EGFP RNAi reverse	TGCTCAGGTAGTGGTTGTCG
EGFP T7 forward	*TAATACGACTCACTATAGG*GACGTAAACGGCCACAAGTT
EGFP T7 reverse	*TAATACGACTCACTATAGG*TGCTCAGGTAGTGGTTGTCG

Italics denote T7 promoter sequences.

Afterward, total RNA was extracted from ISE6 cells. To extract total RNA, one well containing 2.5 × 10^5^ ISE6 cells from a 48-well plate was harvested and transferred to a 1.5 ml tube. The cells were centrifuged at 630 × *g* for 3 min, wherein the supernatant was removed. The RNA was extracted using TRI Reagent (Molecular Research Center, Cincinnati, OH, USA) following the manufacturer’s protocol. cDNA was synthesised from 1 μg of total RNA using ReverTra Ace Master Mix (Toyobo, Osaka, Japan) following the manufacturer’s protocol.

### RT-PCR analysis

Total RNA was extracted from representative wells using TRI Reagent (Molecular Research Center), and cDNA was synthesised using a ReverTra Ace synthesis kit (Toyobo) following the manufacturer’s protocol. RT-PCR was subsequently performed using Hot Start® PCR Mix (Jena Bioscience, Jena, Germany) following the manufacturer’s protocol, using *16S* rRNA as a loading control, followed by specific primers for *FER1*, *FER2*, or *IRP* genes. The PCR profile for *FER1*, *FER2*, and *16S* was as follows: an initial denaturation step at 98 °C for 8 min; 25 cycles of the denaturation step at 98 °C for 30 sec; an annealing step of 65 °C for 60 sec; and an extension step at 72 °C for 60 sec. For *IRP*, the PCR profile was as follows: an initial denaturation step at 98 °C for 8 min; 40 cycles of the denaturation step at 98 °C for 30 sec; an annealing step of 65 °C for 60 sec; and an extension step at 72 °C for 90 sec. PCR products were run on 1.5% agarose gel and stained with ethidium bromide for 50 min and viewed using ATTO system WUV-M20 (ATTO, Tokyo, Japan).

### Ferrous sulphate treatment of ISE6 cells

ISE6 cells were seeded in a 48-well plate of 250 μl of 0.5 × 10^6^ cells/ml and incubated at 34 °C overnight. The culture medium was removed, and the cells were treated with several concentrations of ferrous sulphate (0, 2, 10, and 20 mM) in culture medium at 34 °C at different time points (0, 12, 24, and 48 h).

### *In vitro* ISE6 cell proliferation and survival assays

After ferrous sulphate treatment, ISE6 cells were used for *in vitro* cell proliferation and survival assays. After washing with phosphate buffered saline (PBS), the cells were diluted in 200 μl of culture medium. One hundred microliters of cells was transferred to a 96-well plate for cell proliferation assay (MTT assay) and 1.5 ml tubes for cell survival assay (Trypan blue assay).

The cells in a 96-well plate for were incubated at 34 °C overnight before being subjected to an MTT assay using Cell Titer 96® Non-Radioactive Cell Proliferation Assay Kit (Promega, Madison, WI, USA). Briefly, 15 μl of dye solution was added to each well. The plate was further incubated for 4 h. Then, 100 μl of solubilisation solution/stop mix was added to each well and incubated at 34 °C overnight. Absorbance at 570 nm was determined using a microplate reader (SH-9000 Lab, Corona Electric, Ibaraki, Japan). Ten microliters of cells in a 1.5 ml tube for Trypan blue assay was mixed with the same volume of Trypan blue. The ratio of stained to unstained cells was determined using a haemocytometer.

### Western blotting of ISE6 cell lysates

Ferrous sulphate–exposed cells from representative wells were placed in PBS and bath sonicated for 5 min, amplitude 45 (AS ONE, Osaka, Japan), and then centrifuged at 20,600 × *g* at 4 °C for 5 min. Seventy microliters of supernatant was collected. Equal amounts of supernatant and  2 × loading buffer were mixed and placed in boiling water for 5 min, followed by centrifugation at 20,600 × *g* at 4 °C for 5 min. The protein extracts were separated by 12% SDS-PAGE and transferred to a polyvinylidene difluoride membrane (PVDF) (Millipore, Bedford, MA, USA). The membrane was blocked overnight with 3% skim milk in PBS with 0.05% Tween 20 (PBS-T) and then incubated with primary antibody using mouse-anti *H. longicornis* ferritin (HlFER1) sera (1:250)^8^. An antibody previously prepared for β–tubulin was used as a control^30^. It was incubated at 37 °C for 1 h. After incubation with horseradish peroxidase (HRP)-conjugated goat anti-mouse IgG (1:50,000 dilution; Dako, Glostrup, Denmark) at 37 °C for 1 h, signals were detected using the ECL Prime detection reagent (GE Healthcare, Buckinghamshire, UK) and analyzed using FluorChem FC2 software (Alpha Innotech, Santa Clara, CA, USA). To be able to determine whether the detected signals using the HlFER1 or β–tubulin antibodies from *H. longicornis* would correspond to the FER1 and β–tubulin of *I. scapularis*, the molecular weights of the bands detected were measured using FluorChem FC2 software (Alpha Innotech). The detected band for FER1 at approximately 20 kDa corresponds to the predicted 19.7 kDa MW of FER1 using the ExPASy software (https://web.expasy.org/cgi-bin/compute_pi/pi_tool) (Fig. S2). On the other hand, the approximately 49 kDa MW of β–tubulin corresponds to the predicted 49.9 kDa MW of *I. scapularis* β–tubulin (Accession no. XP_002406661.1) on which BLAST analysis shows that it has a 100% identity with the *H. longicornis* β–tubulin (Accession no. BAK41866.1). For further confirmation of the reactivity of the HlFER1 antibody with *I. scapularis* FER, the knockdown of ISE6 cells with subsequent exposure to ferrous sulphate was performed, proceeded by the Western blotting of cell lysates. No band corresponding to FER1 was observed in the knockdown group, indicating that the FER1 band detected by the HlFER1 antibody is also the FER1 of ISE6 cells (Fig. S3).

### Immunostaining of ISE6 cells

Ten microliters of cells was placed on chamber slides and allowed to air dry. The cells were then fixed with methanol for 10 min. Cells for immunostaining were washed with PBS. Cells were blocked for 1 h with 5% skim milk in PBS at room temperature; afterward, cells were incubated with a 1:50 dilution of mouse anti-HlFER1sera at room temperature for 1 h. Normal mouse serum was used as a negative control at the same dilution. Slides were then washed with PBS and incubated with Alexa Fluor 488-conjugated goat anti-mouse IgG (1: 1,000; Invitrogen, Carlsbad, CA, USA) at room temperature for 1 h. Following washes with PBS, the cells were mounted with VECTASHIELD with DAPI (Vector Laboratories, Burlingame, CA, USA). Images were taken using a confocal fluorescence microscope mounted with LSM 700 (Carl Zeiss, Jena, Germany).

### Ferrozine assay of ISE6 cells

The ferrozine assay for measuring non-haem iron was adapted to determine the concentration of iron in ISE6 cells^31^. After knockdown and/or iron exposure, cells were collected, and cell lysates were collected using the method described above. Concentrated HCl (99.5%) was added and then heated to 95 °C. After cooling to room temperature, the mixture was centrifuged, and the supernatant was obtained, to which was added 75 mM ascorbate or water. Afterward, 10 mM ferrozine (Sigma-Aldrich, St. Louis, MO, USA) was added. Saturated ammonium acetate was added to facilitate colour development. Absorbance was measured at 550 nm, and the iron concentration was calculated based on a molar extinction coefficient of the iron-ferrozine complex of 27,900 M^−1^cm^−1^ and based on the protein concentration. The protein concentration was measured using a Micro BCA Protein Assay Kit (Thermo Fisher Scientific, Rockdord, IL, USA). The total iron concentration is computed from samples with ascorbate. The ferrous iron concentration was computed from samples without ascorbate (reducing agent)^18,32,33^, while the ferric iron concentration is computed from the difference between the total iron and ferrous iron concentration.

### RNA interference (RNAi) using double-stranded RNA with lipofectin to ISE6 cells

RNA interference using double-stranded RNA (dsRNA) was performed to determine the effect of FER and IRP on ISE6 survival and proliferation upon exposure to different doses of ferrous sulphate. The PCR primers used for the synthesis of dsRNA are listed in Table 7. The *FER1* and *IRP* fragments were amplified by PCR from ISE6 cDNA using oligonucleotides, including ISFER1 T7 forward with ISFER1 RNAi reverse and ISFER1 T7 reverse with ISFER1 RNAi forward primers, and ISIRP T7 forward with ISIRP RNAi reverse and ISIRP T7 reverse with ISIRP RNAi forward primers, to attach the T7 promoter recognition sites on both forward and reverse 5′ends. *EGFP* gene fragment was amplified from *pEGFP* by PCR using oligonucleotides containing EGFP T7 forward and EGFP T7 reverse primers as well. PCR products were gel extracted and purified using a QIAquick Gel Extraction Kit (Qiagen, Hilden, Germany). The T7 RiboMAX Express RNAi System (Promega, Madison, WI, USA) was used to synthesised dsRNA by *in vitro* transcription. The successful construction of dsRNA was confirmed by running 1 μL of the dsRNA products in 1.5% agarose gel in a TAE buffer.

ISE6 cells were seeded in a 48-well plate of 250 μl of 0.5 × 10^6^ cells/ml and incubated at 34 °C overnight. The double-stranded RNA (400 ng/well), 15 μl of Opti medium, and 2.8 μl HilyMax (transfection reagent) were mixed and incubated at room temperature for 15 min. dsEGFP was used as a control. Cells in the plates were washed twice with PBS, and then 250 μl of culture media without FBS was added. The previously incubated dsRNA mixture was added to the medium in each well and incubated at 34 °C for 16 h. Two hundred and fifty microliters of the culture medium with FBS was added and incubated further at 34 °C for 32 h. The transfected cells in some wells were harvested, and knockdown was confirmed using RT-PCR as previously described. Remaining knockdown wells were exposed to ferrous sulphate as previously described. Primers used in this study are shown in Table 7. Proliferation ability and survival were determined using MTT and Trypan blue assay as previously described.

### Statistical analysis

Statistical analyses were performed using STATA15.0 software. The data were initially checked for normality and homogeneity assumptions using the Shapiro-Wilk W test for normality and Breusch-Pagan/Cook-Weisberg test for heteroskedasticity. A one-way analysis of variance (ANOVA) or a Kruskall-Wallis test with Bonferonni multiple comparison tests was applied when appropriate. Statistical significance was set as ^*^P < 0.05. Sample sizes were based on our previous results^7^. Sample sizes and reproducibility are indicated in the figure or table legends.

## Electronic supplementary material

Supplementary Information
