# Supplementary material for: Induction of intracellular ferritin expression in embryo-derived Ixodes scapularis cell line (ISE6)
Source: Sci Rep. 2018 Nov 8;8:16566. doi: 10.1038/s41598-018-34860-3 (PMC6224502; doi:10.1038/s41598-018-34860-3)
Supplement: Supplementary file 1 — Supplementary Information [file 41598_2018_34860_MOESM1_ESM.pdf]

Supplementary Information

Induction of intracellular ferritin expression in embryo-derived *Ixodes scapularis* cell line (ISE6)

Authors:

Emmanuel Pacia Hernandez, Kodai Kusakisako, Melbourne Rio Talactac,

Remil Linggatong Galay, Kentaro Yoshii, Tetsuya Tanaka

Table S1. Results of Breusch-Pagan/ Cook-Weisberg test for tests for homogeneity and Shapiro-Wilk W test for normality of data on mortality of cells exposed to different concentrations of ferrous sulphate at different time points

Exposure time (h)	Mortality (*P*-value)	
	Homogeneity	Normality
0	0.1783	0.62321
12	0.3792	0.56886
24	0.8426	0.14889
48	0.1235	0.12764

Table S2. Results of the One-way Analysis of Variance on mortality of cells exposed to different concentrations of ferrous sulphate at different time points (df = 11)

Exposure time (h)	Mortality	
	F-value	*P*-value
0	74.70	0.0001*
12	280.22	0.0001*
24	2307.22	0.0001*
48	2799.76	0.0001*

*Asterisks indicate *P*-values of < 0.05.

Table S3. Results of Bonferronic multiple comparison tests on mortality of cells exposed to different concentrations of ferrous sulphate at different time points

Pairs	Time of Exposure (h)			
	0 (*P*-value)	12 (*P*-value)	24 (*P*-value)	48 (*P*-value)
0 mM vs. 2 mM	0. 004*	0.001*	0.001*	0.001*
0 mM vs. 10 mM	0. 001*	0.001*	0.001*	0.001*
0 mM vs. 20 mM	0. 001*	0. 001*	0.001*	0.001 *
2 mM vs. 10 mM	0. 003*	0. 001*	0.001*	0.001 *
2 mM vs. 20 mM	0. 001*	0. 001*	0.001*	0.001 *
10 mM vs. 20 mM	0. 118	0. 008*	0.001*	0.056

*Asterisks indicate *P*-values of < 0.05.

Table S4. Results of Breusch-Pagan/ Cook-Weisberg test for tests for homogeneity and Shapiro-Wilk W test for normality of data on mortality of cells exposed to different concentrations of ferrous sulphate at 48h

Mortality (*P*-value)	
Homogeneity	Normality
0.9814	0.79068

Table S5. Results of the One-way Analysis of Variance on mortality of cells exposed to different concentrations of ferrous sulphate at 48 h (df = 11)

Mortality	
F-value	*P*-value
44.62	0.0001*

*Asterisks indicate *P*-values of < 0.05.

Table S6. Results of Bonferronic multiple comparison tests on mortality of cells exposed to different concentrations of ferrous sulphate at different time points

Pairs	Mortality (P-value)
0 mM vs. 0.1 mM	0. 416
0 mM vs. 1 mM	0. 004*
0 mM vs. 2 mM	0. 001*
0.1 mM vs. 1 mM	0. 059
0.1 mM vs. 2 mM	0. 001*
1 mM vs. 2 mM	0. 004*

*Asterisks indicate P-values of < 0.05.

Table S7. Results of Breusch-Pagan/ Cook-Weisberg test for tests for homogeneity and Shapiro-Wilk W test for normality of data on ferrous iron concentration on knockdown cells exposed to different concentrations of ferrous sulphate

Ferrous sulphate concentration (mM)	Ferrous iron concentration (P-value)	
	Homogeneity	Normality
0	0.9980	0.68996
0.1	1.0000	0.98616
1	0.9920	0.87508
2	0.9995	0.94201

*Asterisks indicate P-values of < 0.05.

Table S8. Results of the One-way Analysis of Variance on ferrous iron concentration on knockdown cells exposed to different concentrations of ferrous sulphate (df=3)

Ferrous sulphate concentration (mM)	Ferrous iron concentration	
	F-value	***P*-value**
0	1.61	0.2763
0.1	2.15	0.1977
1	50.56	0.0002*
2	39.79	0.0003*

*Asterisks indicate *P*-values of < 0.05.

Table S9. Results of Bonferroni tests on ferrous iron concentration on knockdown cells exposed to different concentrations of ferrous sulphate

Pairs	Ferrous sulphate concentration (mM)	
	**1**	**2**
dsFER vs dsEGFP	0.001*	0.001*
dsIRP vs dsEGFP	0.132	1.000
dsIRP vs dsFER	0.001*	0.001*

*Asterisks indicate *P*-values of < 0.05.

Table S10. Results of Breusch-Pagan/ Cook-Weisberg test for tests for homogeneity and Shapiro-Wilk W test for normality of data on ferric iron concentration on knockdown cells exposed to different concentrations of ferrous sulphate

Ferrous sulphate concentration (mM)	Ferric iron concentration (*P*-value)	
	**Homogeneity**	**Normality**
0	0.9980	0.68996
0.1	0.9958	0.96814
1	0.9967	0.94055
2	1.0000	0.33337

Table S11. Results of the One-way Analysis of Variance on ferric iron concentration on knockdown cells exposed to different concentrations of ferrous sulphate (df=3)

Ferrous sulphate concentration (mM)	Ferric iron concentration	
	F-value	***P*-value**
0	0.38	0.7003
0.1	11.10	0.0096*
1	24.93	0.0012*
2	329.24	0.0001*

*Asterisks indicate *P*-values of < 0.05.

Table S12. Results of Bonferroni tests on ferric iron concentration on knockdown cells exposed to different concentrations of ferrous sulphate

Pairs	Ferrous sulphate concentration (mM)		
	0.1	1	2
FER vs EGFP	0.078	0.006*	0.001*
IRP vs EGFP	0.010*	0.483	0.001*
IRP vs FER	0.409	0.002*	0.001*

*Asterisks indicate *P*-values of < 0.05.

Table S13. Results of Breusch-Pagan/ Cook-Weisberg test for tests for homogeneity and Shapiro-Wilk W test for normality of data on mortality on knockdown cells exposed to different concentrations of ferrous sulphate

Concentration	Mortality (*P*-value)	
	Homogeneity	Normality
0	0.1824	0.36231
0.1	0.1360	0.16292
1	0.5300	0.85874
2	0.5432	0.01793*

*Asterisks indicate *P*-values of < 0.05.

Table S14. Results of the Kruskal-Wallis test on mortality of knockdown cells exposed to different concentrations of ferrous sulphate (df=3)

Concentration	Mortality	
	Chi^2 -value	***P*-value**
0	3.289	0.1931
0.1	5.422	0.0665
1	5.600	0.0608
2	7.200	0.0273*

*Asterisks indicate *P*-values of < 0.05.

Table S15. Results of Bonferroni tests on mortality of knockdown cells exposed to different concentrations of ferrous sulphate

Pairs	Ferrous sulphate concentration (mM)
	**2**
FER vs EGFP	0.0899
IRP vs EGFP	0.0899
IRP vs FER	0.0036*

*Asterisks indicate *P*-values of < 0.05.

Table S16. Results of Breusch-Pagan/ Cook-Weisberg test for tests for homogeneity and Shapiro-Wilk W test for normality of data on proliferation of knockdown cells exposed to different concentrations of ferrous sulphate

Ferrous sulphate concentration (mM)	Proliferation (*P*-value)	
	Homogeneity	Normality
0	0.9966	0.22624
0.1	0.9997	0.91313
1	0.9963	0.11099
2	0.9869	0.06422

Table S17. Results of the One-way Analysis of Variance on proliferation of knockdown cells exposed to different concentrations of ferrous sulphate (df=3)

Ferrous sulphate concentration (mM)	Proliferation	
	F-value	*P*-value
0	8.11	0.0197*
0.1	27.86	0.0009*
1	276.53	0.0001*
2	5630.65	0.0001*

*Asterisks indicate *P*-values of < 0.05.

Table S18. Results of Bonferroni tests on proliferation of knockdown cells exposed to different concentrations of ferrous sulphate

Pairs	Ferrous sulphate concentration (mM)			
	0	0.1	1	2
FER vs EGFP	0.031*	0.003*	0.001*	0.001*
IRP vs EGFP	0.052	1.000	0.025*	0.001*
IRP vs FER	1.000	0.002*	0.001*	0.001*

*Asterisks indicate *P*-values of < 0.05.

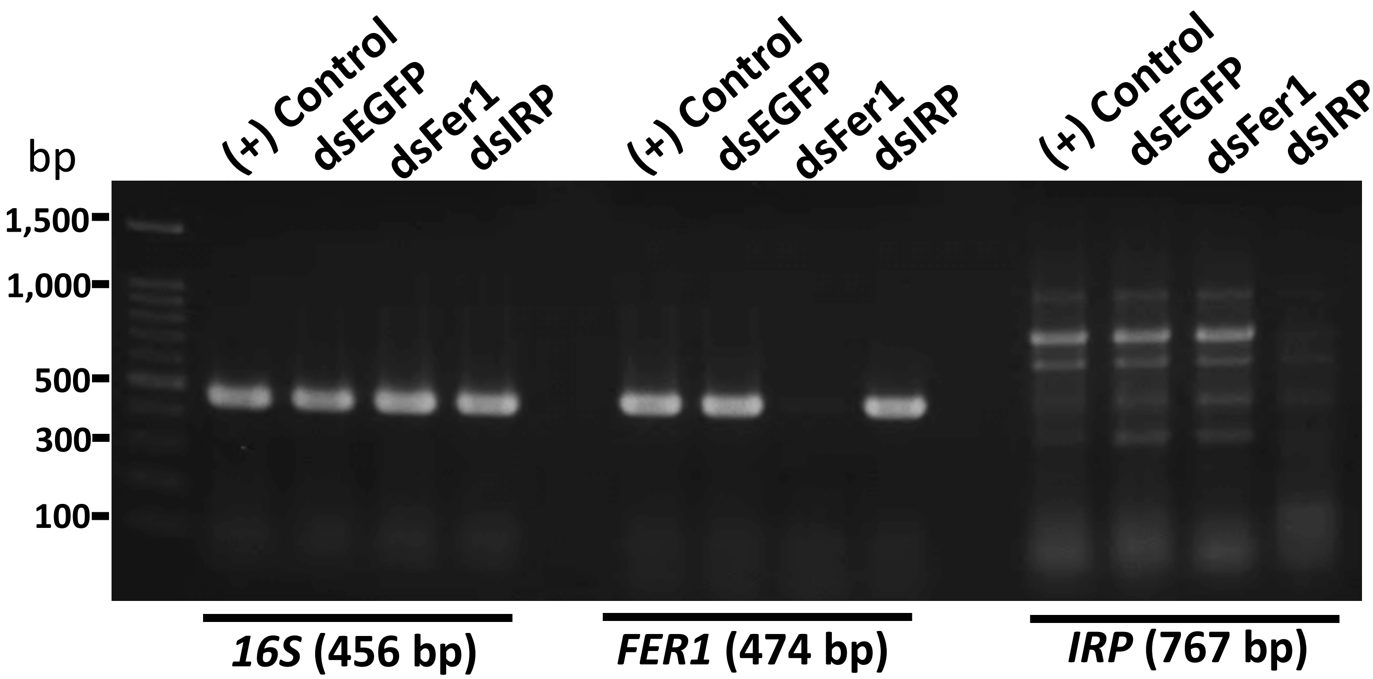

Fig. S1. Gene expression analysis of knocked down ISE6 cells using RT-PCR. Total RNA was extracted from ISE6 cells transfected with dsRNA. cDNA was synthesised and subjected to RT-PCR. PCR products were run on 1.5% agarose gel and stained with ethidium bromide. 16S rRNA was used as a loading control.

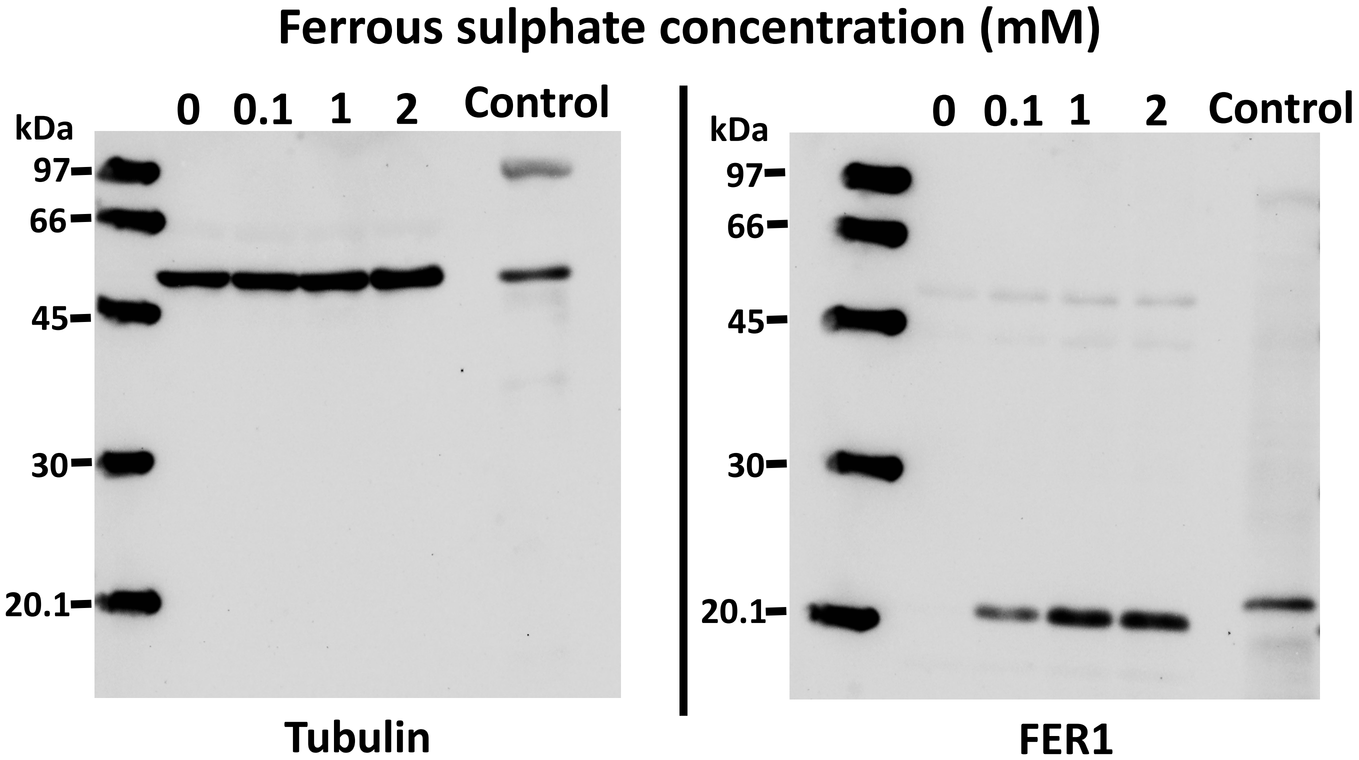

Fig. S2. Full picture of Western blot analysis of tubulin and FER1 protein expression of ISE6 cells exposed to different concentrations of ferrous sulphate (0, 0.1, 1, and 2 mM). The leftmost lane indicates molecular weight markers.

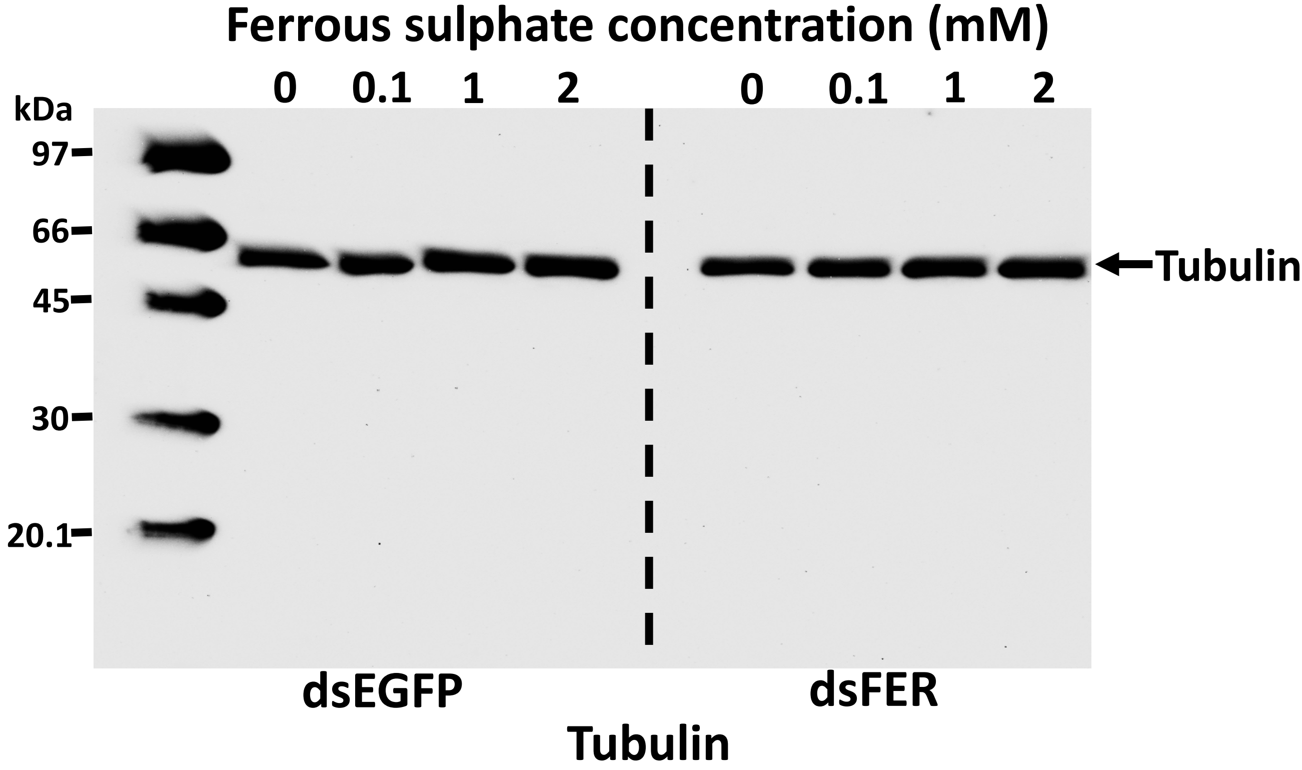

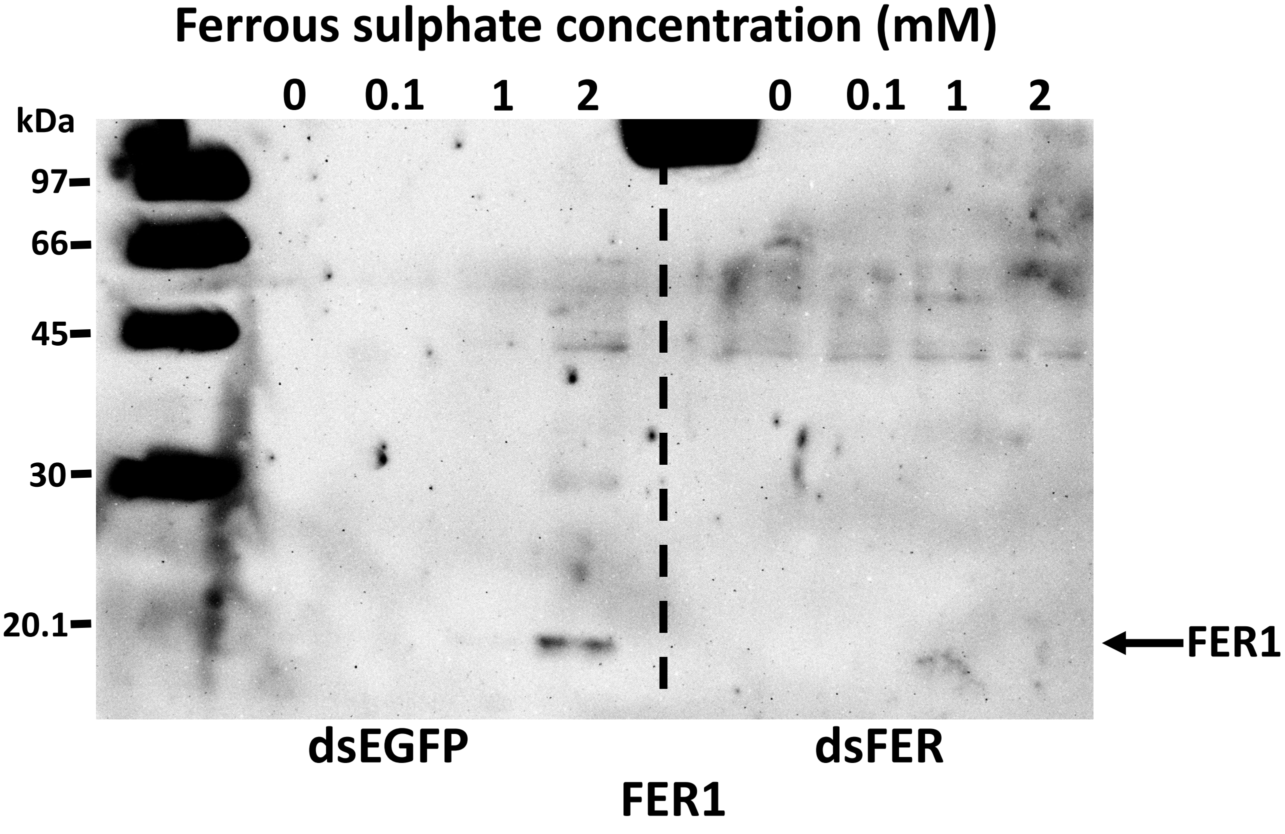

Fig. S3. FER1 expression analysis of knocked down ISE6 cells using Western blotting. Anti-mouse tubulin was used as a loading control.

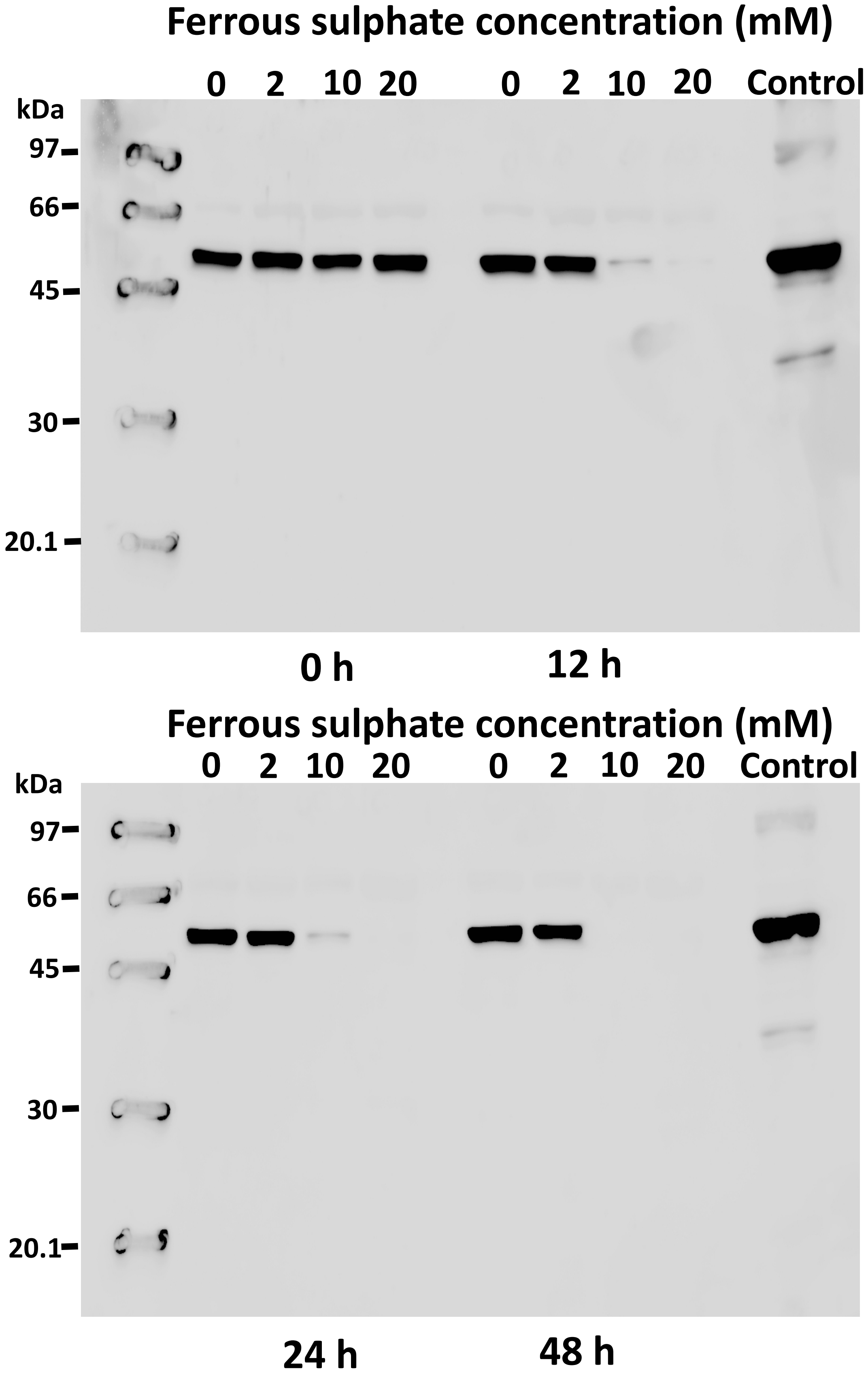

Fig. S4 A. Full picture of Western blotting for tubulin of ISE6 cells exposed to different concentrations of ferrous sulphate at different time points.

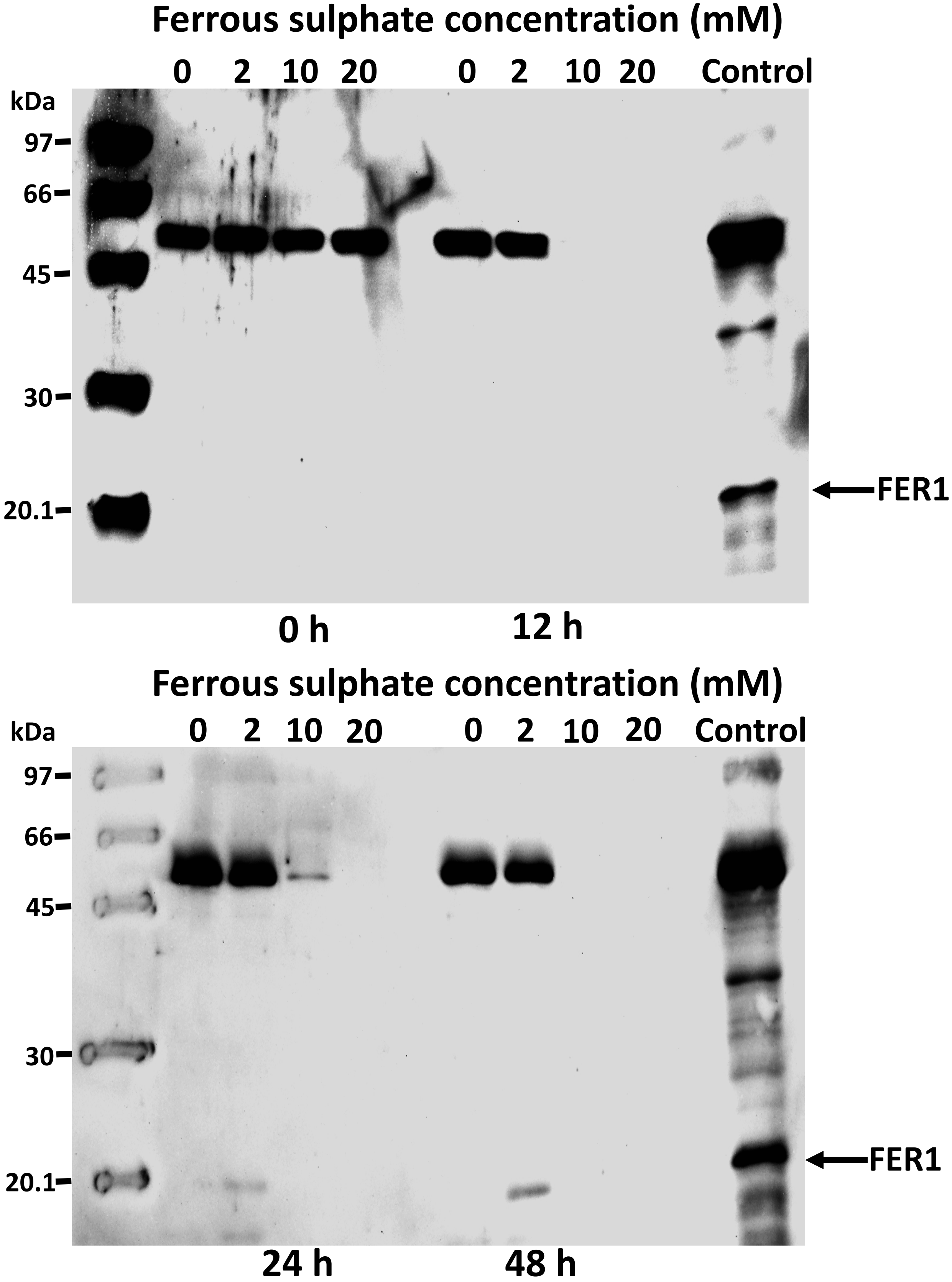

Fig. S4 B. Full picture of Western blotting for FER1 of ISE6 cells exposed to different concentrations of ferrous sulphate at different time points. The same membranes were used after stripping with stripping solution (Wako) following the manufacturer's instruction.

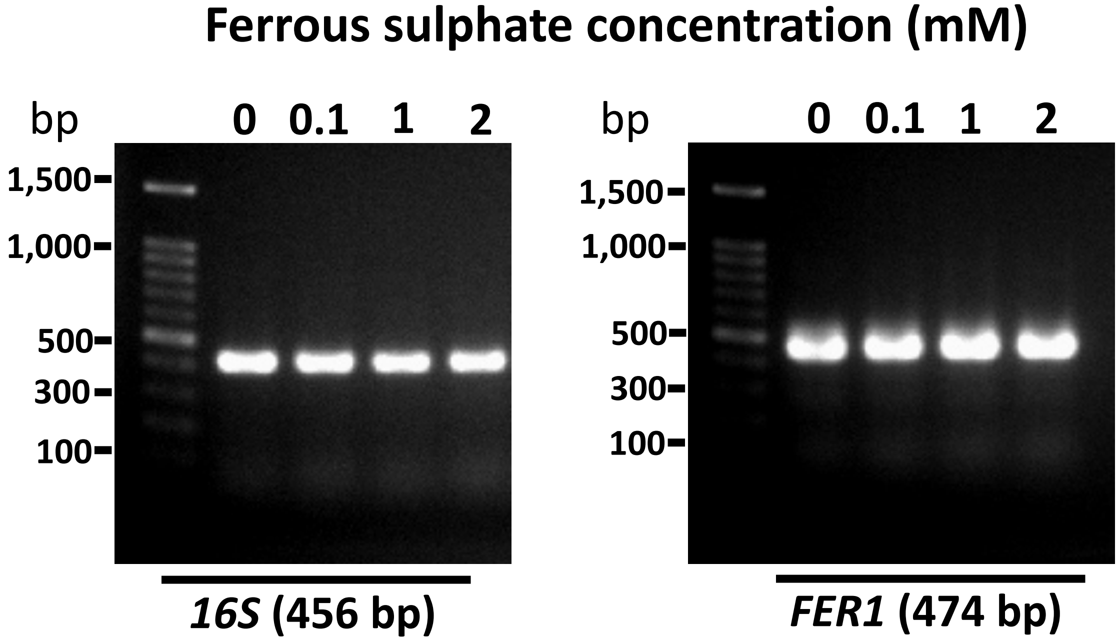

Fig. S5. Full-length gel of *FER1* gene expression of ferrous sulphate (0, 0.1, 1, and 2 mM)–exposed cells for *FER1* was observed using RT-PCR. 16S rRNA was used as a loading control.
